# Developing a programme theory of implementing patient-reported outcome measures for older people living with severe frailty: a mixed methods study using the consolidated framework for implementation research

**DOI:** 10.1186/s41687-025-00951-9

**Published:** 2025-10-24

**Authors:** Faith D. Howard, Jenny Harris, Richard Green, Joy R. Ross, Caroline Nicholson

**Affiliations:** 1 Department of Health Sciences, School of Health Sciences, University of Surrey, Kate Granger Building, Priestley Road, Guildford, GU2 7YH UK; 2 St Christopher’s Hospice, London, UK; 3 Hull York Medical School, Hull, UK

**Keywords:** Frailty, Aged, Patient-reported outcome measures, Implementation, Questionnaire, Patient-centred care, Palliative care

## Abstract

**Background:**

Older people with severe frailty (OPWSF) have palliative needs but typically do not receive specialist palliative care (SPC). Patient-Reported Outcome Measures (PROMs) may offer valuable means to capture these needs. There is a limited understanding of what to include and how to implement PROMs in settings where this group receive most care. The study aimed to: (1) Critically examine how existing PROMs are currently implemented with OPWSF within a SPC setting. (2) Understand how the items with the PROMs are used (3) Develop a programme theory to determine how PROMs can be optimally designed and implemented to effectively capture the needs and priorities of OPWSF in the care setting where they receive most care.

**Methodology:**

Mixed methods study in SPC community service in an urban area in the UK including: • Healthcare professionals (HCPs) providing care to OPWSF with a minimum of 6 months experience in a patient-facing role were purposively sampled: semi-structured interviews (n11); non-participatory observations (n10) - thematically analysed. • One-year retrospective case-note review of 357 episodes of care involving service-users identified with frailty at referral. Exploratory descriptive statistics were used to analyse the use of the Integrated Palliative Outcome Scale (IPOS) alongside additional clinical-led outcome measures. • Date integration using the Consolidation Framework for Implementation Research (CFIR) to develop a programme theory.

**Results:**

PROMs can be effectively used with OPWSF, yet existing PROMs require adapting to ensure they capture the needs that matter most. • Completion of PROMs for this group largely depends on the assistance of others. • HCPs’ use of PROMs may be driven by immediate care and priorities of the care system rather than determining changes over time, with the psycho-social aspect of the PROMs being more challenging to complete.

**Conclusion:**

By utilising the CFIR, the study highlights the complexities and variabilities of using PROMs with OPWSF. Future research should focus on adapting and validating existing PROMs to ensure they are fit for purpose with this population, with the involvement of older people with frailty and unpaid carers. Providers should extend support and training for professionals in the use and value of PROMs and psychosocial-spiritual care.

**Supplementary Information:**

The online version contains supplementary material available at 10.1186/s41687-025-00951-9.

## Background

Global populations are ageing [1]. As people live longer, they are increasingly likely to live with multiple life-limiting conditions, including frailty [2]. Although not an inevitable consequence of ageing, someone with frailty is more vulnerable to decline in physical functioning and reserve [3]. Older people living with severe frailty are more likely to die in a year than those who are not frail, yet are underserved by palliative care [4–6]. Those living with severe frailty, in the last phase of life, prioritise living as well as possible and quality of life rather than managing physical symptoms alone [6]. Effectively capturing and measuring these needs and priorities is essential to support personalised care and wider system response. Patient-Reported Outcome Measures (PROMs) and Patient-Reported Experience Measures (PREMs) are tools to capture care from the patient’s perspective [7, 8]. The use of PROMs in specialist palliative care practice has been shown to improve the identification of needs, enhance communication between the patient and providers and within teams, and improve patients’ symptoms and overall outcomes [9–12].

Most individuals with severe frailty, however, will not receive specialist palliative care and are likely to be cared for in a spectrum of settings where PROMs and PREMs are not routinely used [5, 13]. There is limited evidence of what to include and how to implement PROMs in practice with this group [14–16]. This study aimed to (1) critically examine how existing PROMs and PREMs are currently implemented with older people with severe frailty within a specialist palliative care setting, (2) understand how the items with the PROMs are used, and (3) use this insight to develop a programme theory using a logic model that can guide other care settings in using PROMs to capture the needs and priorities of this population effectively.

## Methods

A three-phase mixed-method design, based in a specialist palliative care community service in an urban area in the UK. This study incorporated the use of semi-structured interviews and non-participant observations with purposely sampled Healthcare professionals (HCPs) (detail in phase 1 below), a retrospective case-note review of PROM and key clinical measurement tools routinely collected at each visit (phase 2), and the integration and development of the logic model (phase 3). Analysis was initially conducted separately for phases one and two and then integrated, using the ‘*following the thread*’ framework [17].

The mixed method approach utilised was based on *triangulation* reasoning, where different types of data on the same subject of interest are combined to provide a more comprehensive answer to the research question [17].

The development of a programme theory facilitates an evidence-based understanding of an innovation (in this case, a PROM) visually through a logic model which represents the relationships between resources, activities, outputs, and the outcomes and the impacts of those activities [18]. The study was guided by the Medical Research Council’s (MRC) framework for developing complex interventions [19], and the Consolidated Framework Implementation Research (CFIR) was used to critically assess the factors that impact the use of PROMs and PREMs with this group [20]. The CFIR is a widely used and practical implementation science approach, based on five key elements: the innovation, inner settings, outer settings, individuals involved in the intervention. Given the action-oriented aims of the study, using CFIR enabled a richer understanding of the complexity of these factors [21, 22].

### Ethical approval and public and patient involvement and engagement

Guidance from Public and Patient Involvement and Engagement (PPIE), which included caregivers of older people with frailty, was sought. The group informed the design of the non-participatory observations. This resulted in an enhanced procedure that informed service-users and caregivers of the research activity taking place and ensured there were mechanisms to allow them to decline the researcher’s presence for their consultation. Ethical approval was received from the University of Surrey’s Research Ethics Committee (ref: FHMS 22–23 155 EGA 31 May 2023).

#### Phase 1: Observations and semi-structured interviews

##### Sample and procedures

HCPs providing care to older people with severe frailty with a minimum of 6 months experience in a patient-facing role in a palliative care setting were purposively sampled. Non-participant observations and semi-structured interviews were conducted (by FH) between August and October 2023, each method requiring separate consent from participants.

Non-participant observations with HCPs took place during consultations with older people with severe frailty in their home and while HCPs were documenting consultations. Where observations involved patients and their family members, the HCP informed them of the research activity and patients and family members chose whether the researcher could be present. Using a standardised proforma allowed for consistency of data collection across observations in relation to the PROM use as part of the HCPs assessments (Supplementary data file 1) [23]. This was supported by in-depth researchers’ notes to inform reflexivity [24].

Interviews were conducted either at the HCP’s place of work or via Microsoft Teams and recorded via Microsoft Teams or an encrypted audio recording device and transcribed. They were conducted concurrently with the observations. The interview topic guide was designed to support reflexivity between the two research activities. It was developed collaboratively within the research team, reviewed by a palliative care HCP to ensure the terms used were familiar to participants, and piloted with a clinical academic before data collection (Supplementary data file 2).

Interviews (*n* = 11) and observations (*n* = 10) were thematically analysed, supported by Nvivo12 software [25]. Using an inductive approach, initial codes and themes were developed after familiarisation with the transcripts and field-notes (by FH). Given the implementation approach of the study, additional codes were drawn from the CFIR, and some subcodes were changed following discussions as a team.

#### Phase 2: Case-note review

##### Sample and procedures

Eligible records for the review were service-users aged 65 or over with frailty and referred to the community palliative care service between Dec 6th 2022, and Dec 6th 2023. Anonymised retrospective routine data from case-notes was extracted by the hospice and examined to understand PROM collection and the context in which PROMs were used. In addition to the PROM, Integrated Palliative Outcome scale (IPOS), IRR; k = 0.65-85 [26, 27], clinical measurement tools extracted included the Clinical Frailty Scale (CFS), IRR; k = 0.80 [28] Phase of Illness, IRR; k = 0.61–0.77 [29]; Australian Karnofsky Performance Scale (AKPS), IRR: ICC = 0.9 [30]. Data points included demographics, details of the outcome measures used, including PROMs and, for a sub-sample (*n* = 12), textual notes from three additional qualitative data points and the Views-on-Care experience measure [31]. See details of extracted data points in supplementary data file 3.

Exploratory descriptive data analysis was conducted in R studio. R code was written by FH and checked by JH. Textual notes from the subsample were tabulated using a framework analysis supported by a conceptual framework of the palliative care needs of those with frailty [32, 33].

#### Phase 3: Integration and development of the logic model

FH conducted the integration, supported by discussions within the research team (CN, JH, RG, and JR). Reflexivity was central to this process, which required active engagement from the researcher to develop empirical evidence [34]. Given that the action-orientation study aimed to detail the programme theory, the CFIR supported data integrations to enable insight for other settings considering implementing PROMs with those caring for people with older people with frailty. (see Supplementary file 4). The CFIR guided the integration process, using reporting guidelines [20].

The “Following the thread” technique was used, which involves identifying findings in one data source and then exploring whether they appear in another [17]. This method of integration allows for the development of themes that transcend individual data components, maintaining the richness of exploratory qualitative data while integrating the specificity of the quantitative data [35]. This approach is beneficial in implementation science, where the subject matter is complex and multifaceted.

#### Quality

The authors followed the *Good reporting of a mixed-methods study checklist* found within the supplementary data file 5 [36, 37].

## Results

### Phase 1: Observations and semi-structured interviews

#### Participant and observation characteristics

Two of the eleven interviews were conducted virtually using Microsoft Teams, and the remaining were conducted face-to-face at the participants’ workplace (Table 1). In all ten observations, service-users were White British, with clinical frailty scores of 6 (n2), 7 (n6), and 8 (n2). Eight service-users lived alone, and family or formal caregivers were involved in the assessment in all but one encounter. Of the ten clinical encounters, seven were reassessments, and three were first assessments. Seven encounters included conversations around “what matters most” or patient goals. Physical assessments were conducted in all encounters. Practical needs, such as care coordination, were addressed in eight out of ten. Social needs were discussed in seven encounters, while psychological needs were assessed in half. Spiritual care was the least frequently included need, with only two encounters addressing this.

**Table 1:** Interview and observation participant demographics

Participants Characteristics (*n* = 11)	*N*
**Gender**	
Female	10
Male	1
**Ethnicity**	
White British/other	9
Mixed British/other	2
**Occupation**	
Associate Clinical Nurse Specialist	2
Associate Paramedic Specialist	1
Clinical Nurse Specialist	4
Doctor	1
Physician Associate	1
Physiotherapist	2
**Setting**	
Single Point of Assessment via telephone	2
Patient’s own home	9
**Year of Experience in setting**	
6mth-1yr	2
1-5yrs	1
5-10yrs	1
10+	7

Most PROMs were proxy-completed by the HCP following the consultation (*n* = 7). In one case, the PROM was completed before the assessment by the service-user and caregiver. In two cases, the HCP completed the PROM with input from a family caregiver during the consultation. Physical health items were documented in all encounters. Social, psychological and practical items were completed in five cases, and the spiritual item was completed in two cases. Views-on-Care items were not completed in any of the observations.

The PROM was found to be able to identify the breadth of the needs of the older person with frailty. However, the observations and sub-sample of textual case-note also indicate that the PROM may have also missed needs in this group. The physical or cognitive capabilities of those with frailty affected how PROMs were completed, frequently involving the support of others. For HCPs, the PROM appeared to provide a common framework for systematically capturing needs across the organisation. The qualitative questions at the start of the tool seemed to be most valued by HCPs, which could provide context to the rest of the items within the measure. However, workforce pressures impacted how PROMs were used, with variation observed. There appeared to be greater confidence in scoring physical items, where HCPs could use a clinical-led approach to support assessment. There was a sense that there was a skill associated with using the PROM to create space/time to ask PROM questions, with the “feeling at peace” item identified as challenging to incorporate into clinical consultations and score within the PROM. For some, if used in isolation, PROMs were seen as a barrier to conducting assessments and communication with patients and seen as a burden. Experience items were particularly perceived as burdensome and were viewed not to enhance care (see Table 2).

**Table 2:** Themes identified within the interviews

Theme	Summary	Evidence
Capturing the needs of Older People with frailty	The PROM was able to identify the breadth of the needs that the older person with frailty may have, yet it also missed aspects specific to this group.	*“my more young people are younger without frailty or single condition*,* their symptoms might be focused on a particular area. It [the IPOS] does show the breadth and*,* therefore*,* the complexity of the support that you’re trying to give and the problems you’re trying to tackle.”In1p1* *“the difficulty is that there’s a bit more to people with frailty that it [IPOS] doesn’t do and it’s where do you put that information” In2p2* *“Frailty often comes with non-cancer related pain… so when people are scoring overwhelmingly on pain that looks like something we should be actively managing*,* but the action plan is to refer to a different team. And that score might never change. But that doesn’t mean it’s not a useful tool. It’s just the sensitivity of it*,* in terms of how we should be responding to it maybe.“In11P11*
Universality in use of the PROM:	The PROM provided a common language to capture needs systematically across the organisation. The qualitative questions at the start of the tool appeared most valued by the professionals, which could provide a context to the rest of the items within the measure. However, time pressures and staff turnover impacted how PROMs were used.	*“It’s a universal tool that we’re all using doctors*,* nurses*,* everyone in the kind of organisation.“In3P3* *“Whereas IPOS*,* perhaps it’s just engrained into the way we work in this organisation*,* but I think it’s really*,* it is a good tool in getting an overall summary” In4P4* *“I think when we ask about what is the most important for you and we try to do something to match that needs is when you are helping them to be in peace with themselves as well.”In7P7* “There is a high turnover of staff… I don’t think it’s routinely retaught. So people are using it, but they are using it how they think it should be used, rather than how it definitely should be used.” In11P11
Involvement of others by necessity	The physical or cognitive capabilities of those with frailty affected how PROMs were completed, frequently involving the support of others.	*“Typically with our older people with frailty*,* there’s more deferment should we say to family members”In1p1* *“…so you are often relying on a lot of collateral history and relying on a lot of observation for that. That can be a bit of a challenge.”In9P9* *“So we’re there for the person*,* but we’re also there for their loved ones that are caring for them… you’re often doing a visit to two people. But it’s not captured.”In5p5* *“There have been some… the response will be 3 and 4s*,* you know this is awful*,* this awful. And actually*,* it’s the carer distress coming through” In9P9*
*Challenges of different items*	There appeared to be greater confidence in scoring physical items, where HCPs could use other clinical skills to support assessment. There was also a sense that there was a skill in creating space/time to ask PROM questions, with the “feeling at peace” item identified as challenging to incorporate into clinical consultations and score within the PROM.	*“[if] I’m doing it afterwards*,* and I’ve done an assessment*,* and I haven’t asked a question around the physical*,* generally I’d score it low because if they haven’t mentioned it. Then I’m figuring it’s not affecting them significantly on that day. I think the psychological are harder to score. You don’t want to say someone isn’t having any*,* you know*,* anxiety or things if they are*,* but it’s not particularly obvious in their behaviour*,* and they haven’t mentioned it”In1P1* *“If you are in peace*,* for*,* and in my experience asking that to someone that is not actively dying*,* it’s a bit tricky question and most of the time they became grumpy with that question. To be honest*,* they don’t understand.”In7P7* *“I think we perceive things as clinicians differently to how people subjectively perceive their problems.”In8P8* *I think psychological issues particularly are so much better addressed face to face because I think that’s particularly key to get the patient themselves talking*,* you know*,* about that and not relying on someone else perception. IN2P2* *“But there is a certain skill*,* and I think you have to work on that… you can’t just think of it as check boxes and going through*,* you’ve got to find*,* we all have our own little ways*,* how do you get into difficult subjects”In2P2* *“Sometimes you get*,* have to go in and do a general conversation and throw those little talks in…”In5P5*
Burden	Motivations regarding whether/how to complete PROM appeared more driven by the individual’s patients’ needs than an organisation’s. For some, if used in isolation, PROMs were seen by as a barrier to conducting assessments and communication with patients and as an added burden to patients. Experience items were particularly perceived as burdensome.	*“I think bringing out any kind of form without a really good reason for doing it can make it*,* feel like it*,* can be a little bit of a barrier to forming that fluid and organic relationship with a patient and their families because you have got a piece of paper that your constantly referring to. But there are ways around that*,* you know.” In11P11* *“I respect a lot of the IPOS… it’s a guide and I think we take the risk to be very attached to the guide and less open to catch What is important for that patient and what are the needs and what are the worries and how we can help them.“In7p7* *“So if I am doing the IPOS with somebody*,* then I’m not doing it [View on Care] at all. I’m not asking them their views on care… then I’m crossing that bit off and saying*,* ‘Just fill it up to there for now.’"In10P10* *“The amount of times people don’t understand*,* know why I’m doing this thing [ipos]. “In1P1*

### Phase 2: Case-note review

#### Characteristics of cases

A total of 4535 service-users over 65 were referred to the hospice at-home service. Of this group, there were 357 episodes of care involving service-users with frailty identified at referral (Table 3). This group’s mean age was 90 (± 7.45 SD), with a median 91, range of 66–107 and an interquartile range (IQR) 86–95. The mean age for all service-users over 65 (including those without frailty) was 82 (± 8.99 SD), with a median of 83 and a range of 65–107.

The mean referral length for those with a diagnosis of frailty identified at referral was 56.9 (± SD70.0), with a median of 30 days, range of 1 to 342, and an interquartile range (IQR) 11.0-78.5. The length of referral analysis could only be conducted on complete referrals (n260), meaning the service-user had either died or completed the intervention. The referral length for those without a recorded discharge status (n97) could not be determined within this analysis. For all those with a diagnosis of frailty, when the IPOS was first recorded, 50.5% (n135) had a CFS value of 7 or higher on referral, 43.4% (116 were not recorded; 21.2% (n73) of these were categorised as being in a stable Phase of Illness, with the remaining cases, being in an unstable, deteriorating or dying Phase of Illness; 62.2%(n186) had an AKPS score of 30% or less (i.e. almost completely bedbound).

#### Implementation of proms

**Fig. 1:**
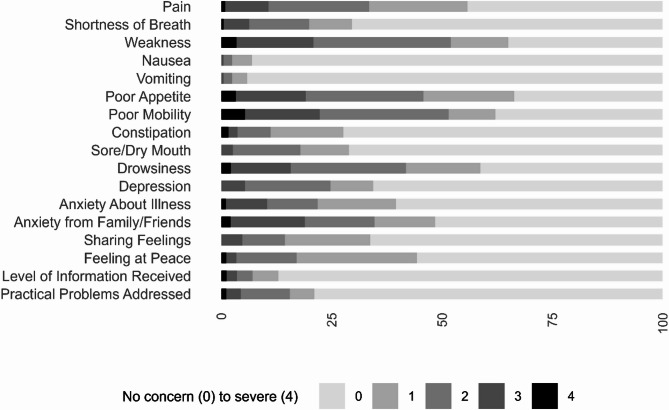
IPOS: Severity of symptom and need for those with frailty diagnosis referral on first assessment

Figure 1 shows the distribution of the IPOS score for this group. Mobility, weakness and appetite were most frequently rated at levels 3 and 4 (severely and overwhelmingly affected) within the physical domain. In the psycho-social domains, the highest proportion of level 3 and 4 scores was for family anxiety, anxiety about illness and depression. Nausea and vomiting were the least reported of all the items.

#### The completion of measurement items recorded on the first assessment

The clinical measures (POI and AKPS) were recorded in all but 3.7% (n13). Within the IPOS, items within the physical domain were reported more frequently (> 88%) than the psycho-social domain (> 49.7 - <55.4%). See Supplementary file for visualisation.

‘Main Concerns’ and ‘*What is important to you right now’* conversations were documented within the free text in 100% of the subsample of 12 records. These conversations were most frequently related to physical (n7) and social (n6) domains. In the physical domain, these included pain, deterioration in mobility and weakness. In the social domain, family support/distress was most frequently documented. The desire for “Being comfortable” or “comfort” (n4) was also frequently documented as a *Main Concern*, though this may have crossed multiple domains.

**Table 3:** Episodes of care for frailty referrals - demographic characteristics

Episodes of care for individuals referred with frailty on referral*:	357	The Average length of referral (days):ꬷ	
		Median Mean (range) SD IQR. 25th & 75th percentile	30 56.9(1-342) ± 70.0 11.0-78.5
Gender: n(%)		Age (years):¶	
Female Male	246 (69.0) 111(31.0)	Median Mean (range) SD IQR. 25th & 75th percentile	91 90 (66–107) ± 7.5 86–95
Ethnicity: n(%)		Social Economic:	
Black Mixed: South Asian: White: Other: Unknown	28(7.8) 19 (5.3) 13 (3.6) 259 (72.5) 6 (1.7) 32 (9.0)	IMD-Rank - Median (range) SD IMD-Decile - Median (range) SD	17075.3 (2640–31879) ± 8537.6 5.6 (1–10) ± 2.6
Diagnosis -primary: n(%)		Diagnosis - secondary: n(%)	
Cancer Cardiac CVA Dementia Frailty Neuro Other Non-Cancer Diagnosis Renal Respiratory	19 (5.3) 27(7.6) 6(1.7) 69(19.3) 125(35.0) 3(0.8) 92(25.8) 4(1.1) 12(3.4)	Cancer Cardiac CVA Dementia Frailty Neuro Other Non-Cancer Diagnosis Renal Respiratory NA	24 (6.7) 10 (2.8) 2 (0.6) 28 (7.8) 207 (58.0) 3 (0.8) 35 (9.8) 2 (0.6) 4 (1.1) 42 (11.8)
1st IPOS Completed by: n (%)		Phase of illness n (%)	
Patient Staff member With help from a friend/relative With help from a staff member	17 (5.9) 218(67.3) 34 (10.5) 55 (17.0)	Stable Unstable Deteriorating Dying – low complexity Dying – high complexity Missing	73 (21.2) 61 (17.7) 156 (45.2) 34 (9.9) 5 (1.4) 13 (3.7)
AKPS n(%) 100 90 80 70 60 50 40 30 20 10 Missing	0 0 0 4(1.3) 9(3.0) 25(8.4) 37 (12.4) 57 (19.1) 112 (37.5) 17 (5.7) 34 (11.4)	Clinical Frailty Scale n(%) 1 2 3 4 5 6 7 8 9 Missing	0 0 0 0 1 (0.3) 15 (5.6) 69 (25.8) 58 (21.7) 8(3.0) 116 (43.4)

* Out of 4535 service-users aged 65 or over patients accessing the whole service

ꬷExcludes cases which did not have a value within the discharge date as an episode of care was still open

¶Service-users over 65 (without frailty on referral): Median 85, Mean (range) 84 (65–106), SD ±8.48; IQR. 25th & 75th percentile: 77–90

Phase of illness values out of 345 1st assessments | AKPS values out of 323 1st assessments | Clinical frailty values out of 267 1st assessments

IPOS (Integrated Palliative Outcome Scale): 5 level scale - No concern (0) to severe (4) [27]. Phase of illness: 5 level scale - Stable; Unable; Deteriorating; dying; Died [29]

AKPS (Australia-modified Karnofsky Performance scale): 11 level scale − 0%: Dead to 100%: No evidence of disease [30].

Clinical Frailty Scale: 9 level scale very fit (1) to Terminally ill [9]

### Phase 3: Integration of findings and development of the logic model

**Fig. 2:**
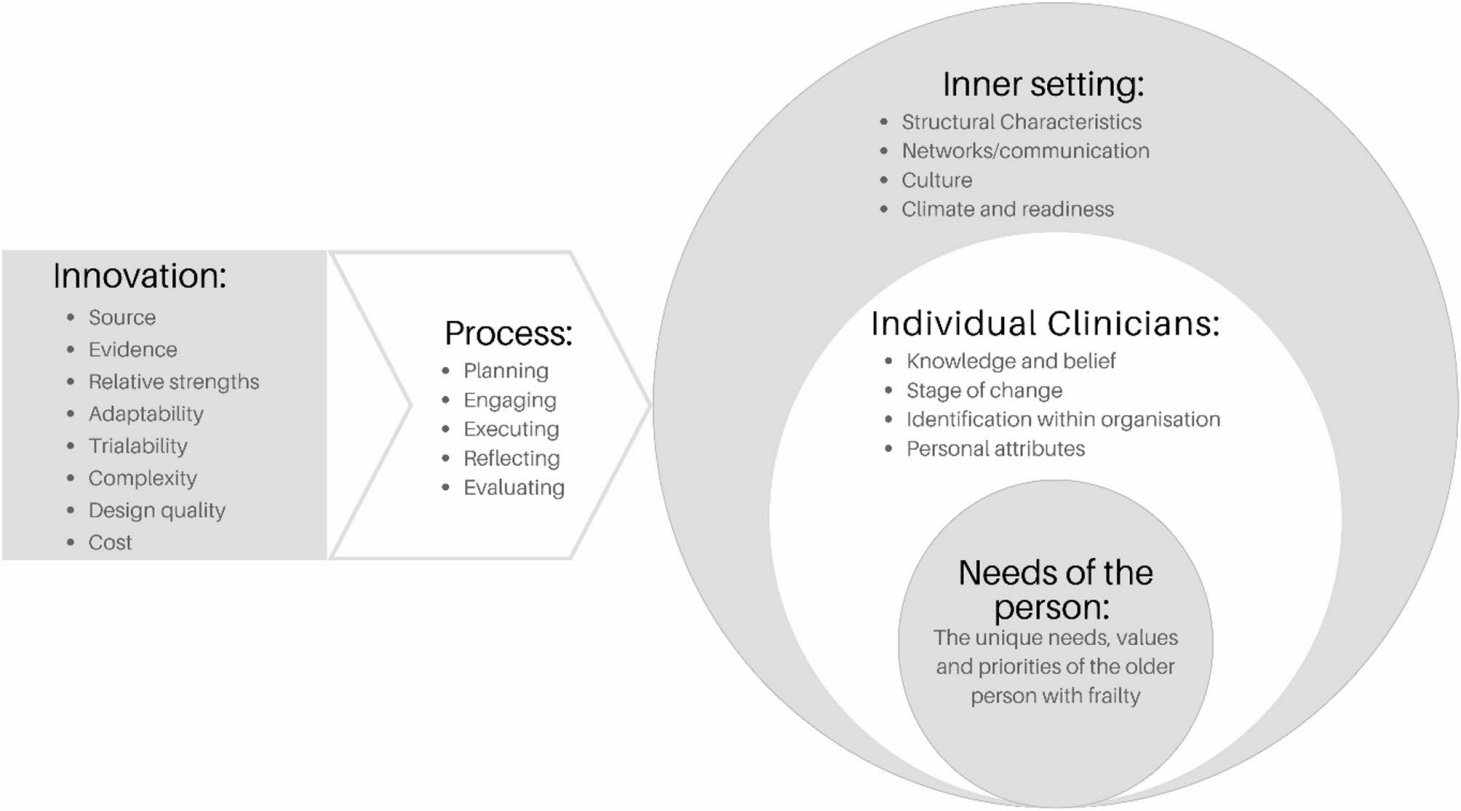
Modified CFIR developed during integration. Adapted from: Damschroder et al. [20]

Themes were identified using the umbrella domains within the CFIR with some modifications (Fig. 2). Notably, the *Outer domain* was not populated within the analysis. In the CFIR, the Outer domain relates to macro-level constructs, such as local and national policies. This absence may be due to the sample frame of participants who were direct care providers rather than strategic leaders. Additionally, we introduced a new domain focusing on the personalised needs of the older person living with frailty. These concerns did not easily align with the existing framework, yet were seen to be central to how the innovation (the PROM) *was* and *could* be used.

#### The innovation: PROM or PREM

For most HCPs, the IPOS was valued as a tool to enable them to identify the breadth of symptoms experienced by someone living with frailty. However, participants also acknowledged that the IPOS placed less emphasis on important concepts, such as specific practical needs related to access to social care. Others questioned the relevance of particular items. For example, the “feeling at peace” was expressed by some participants as being confusing for individuals not considered to be in a dying phase. Other participants identified issues of the sensitivity of physical items, which may have very different aetiology for this population (i.e., chronic generalised pain rather than acute pain due to a specific disease), which required a different individualised and system response.

#### Process: “How the innovation (PROM or PREM is being used)”

Variations were observed in PROM use. Observations found that PROMs were primarily completed by proxy, typically by professionals following consultations. When formal and unpaid caregivers were present, they contributed information during the assessment. Observations and case-note reviews showed that physical aspects of the PROM were completed more frequently than other items. Participants across the sample expressed psycho-social elements of the PROM as more challenging to score. The case-note review found that the AKPS was more consistently used alongside the IPOS than the frailty-specific CFS.

#### Needs of the person: “The older person with frailty”

The case-note review identified that patients entering the service were typically in a “deteriorating” or “unstable” category of the Phase of illness measure (indicating care plans needed ongoing review with expected worsening of current problems). The AKPS and CFS scores indicate that most experienced poor functionality and dependency on others. Among physical concerns, mobility and weakness ranked highest, and family anxiety was a significant psycho-social symptom. Caregiver support was frequently cited as a concern (emerging from in-depth case-note review). Within the observations, practical support for both the service-user and caregivers was a predominant feature of clinical visits. This, however, was not fully reflected within the PROM content, and given the variations in missingness items within the case-note review, meant psycho-social-practical needs were less likely to be captured within the PROM.

#### Individual: “The people using the innovation (the PROM or PREM)”

HCPs were motivated to tailor care to what matters most to their service-users. PROMs served as both barriers and facilitators in the delivery of this. Some HCPs who discussed the role of the PROMs or specific PROM items in the consultation reflected that it acted as a mediator to identify needs. Conversely, others avoided direct use with service-users and caregivers within a consultation, citing concerns about the burden or perception that it could be seen as a “tick-box exercise” if used in isolation. This concept of burden was particularly evident with the “Views on Care” PREM items, which were not obtained during any observations. When explored within interviews, participants expressed uncertainty about how Views on Care items directly benefited the individual’s care.

#### Inner setting: “Where the innovation (PROM or PREM) is being used”

Evidence from the interviews and observations showed that the PROM was used to screen, provide a framework for assessment, document assessments, and facilitate communication between colleagues. The study took place within a post-COVID-19 climate, where participants reflected on workforce pressures, which included increasing complexity of caseload and high staff turnover, and in interviews, reflected on how this impacted the use of the PROM in practice across teams and the organisation, contributing to a variation in use.

**Fig. 3:**
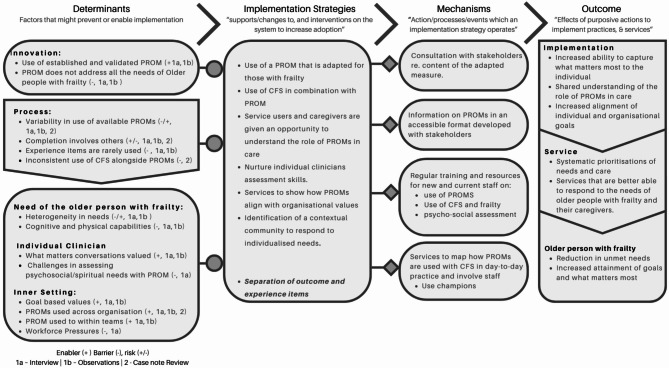
Logic model – using PROMs to capture and measure what matters most to older people with frailty

Building on the CFIR constructs, findings were then used to develop the programme theory illustrated in the logic model in Fig. 3. The logic model comprises five core domains: the Situation, Determinants, Implementation Strategies, Implementation Mechanisms, and Outcomes/Impact, and it illustrates strategies for addressing these determinants and the mechanisms required for implementing these strategies, resulting in measurable outcomes [38]. Supplementary Data File 6 details how the findings from the components of CFIR were mapped onto the determinants at the start of the logic model.

## Discussion

This study contributes to the growing evidence on implementing PROMs within the clinical setting. Developing a programme theory underpinned by CFIR and MRC provided rigour in both the study design and analysis, highlighting the complexity and the contextual factors influencing PROM effectiveness [19] This study identified that overall, HCPs valued the IPOS within this setting, however, there was variability in PROM use. Critically, this study highlights the challenges of using PROMs with older people with severe frailty. Importantly, the programme theory articulates (1) how existing PROMS and PREMs may need adaptation to capture what matters most to older adults with frailty and, (2) how these challenges could be addressed to support the implementation of PROMs in settings where this group receives most of their care.

Most of the people with frailty entering the service were either in a deteriorating or unstable phase of illness, yet there was a wide range and high SD of the length of referral. This does not rule out the possibility that two distinct groups are referred to the service. (1) Those who died or where the intervention is completed within the median of 30 days and (2) a group with a distinctively more extended referral. To understand the latter’s significance, a more extended data collection period would be required. This may reflect the uncertainty associated with prognosis (or time until death), although it does not provide an understanding of patient needs or the care provided. Overall, the IPOS was seen to be a useful outcome measure for this group and a tool to support clinical assessment and team communication. The IPOS is a well-validated and comprehensively tested PROM for people with life-limiting conditions, such as cancer [27, 39, 40] However, this study provides evidence that implementing its use with older adults with severe frailty is challenging. It further supports the case of adapting a PROM specifically for older people with severe frailty [15].

This study’s variability in PROM use and data quality highlights the challenges of collecting and recording PROM data within clinical settings. The origins of these variabilities appear to be multifactorial. People with severe frailty entering the service were universally dependent on others for care, and PROMs were primarily proxy-completed within this study. HCPs’ use of IPOS was driven mainly by the need to inform immediate and individualised care. Service-user burden appeared to be a central consideration in how the PROM was completed, and psycho-social aspects of the PROM, particularly, were more challenging. This was coupled with workforce pressures that contributed to the opportunities for training new and existing staff. While these findings are not generalisable, Bradshaw [12] also identified similar challenges regarding psychological aspects within the IPOS, suggesting this trend is not unique to those with frailty.

Although the IPOS was integrated into routine practice (and electronic service-user records), parts of the IPOS and other clinician-led measures examined within this study were not all utilised to the same extent. Given that HCPs largely completed the PROMs, variation in item response between physical and psychosocial-spiritual items may, in part, be because some HCPs did not distinguish the difference between the IPOS (as PROM) from clinical measurement tools used as part of routine care. Unlike clinical measurement tools (which are typically bio-medical in focus), PROMs are developed to capture need from a person-centred perspective [7, 41]. While the data quality of the Phase of illness and AKPS was excellent, this was less so for the PREM, Views-on-care, and HCP-completed CFS. HCPs perceived the Views-on-care items as lacking a direct connection to improving the individual patient’s care. Further, it is possible that because PREMs are evaluations of the care the HCPs provide, there may be ethical and practical considerations about their validity and implementation, which require examination in future research [42]. The differences in the collection of CFS compared to AKPS also warrant further consideration. The high completion rate of the AKPS (a palliative care-specific measure) indicates successful implementation within this specific specialist palliative care context. Nevertheless, for this population, it may be less meaningful as an indicator of decline for those already affected by poor mobility [43]. However, the inconsistent use of the CFS may suggest a need for improved, broader frailty awareness and guidance on its implementation in this specific setting.

### Implications for practice, policy and research

Care providers should support a shared understanding of the role of PROMs in individualised care, enabling effective facilitation of PROM completion. Older adults living with severe frailty in this last phase of life prioritise living well now over end-of-life needs [6]. The use of “What is important to you” questions in combination with the PROM, as observed within this study, may support both a tailored and capabilities-based approach used in other measures, such as ICE-CAP [44, 45]. Given that caregivers frequently adopt a dual role of translator between services and deliver most day-to-day care to older people with frailty [46], any future PROM implementation also requires input from this group.

HCPs have an ethical and professional responsibility to identify care needs and respond appropriately [47]. This underscores the importance of using PROMs cyclically. Interview participants expressed differences in confidence and opportunities to facilitate psycho-social-spiritual assessment compared to those related to physical needs. Correspondingly, this may also be related to capacity (or ability) to respond to a specific identified unmet need within the service. The professional proxy version of the IPOS contains the *Cannot Assess* box for each item, which can be marked when the proxy cannot assess symptoms due to the patient’s condition; however, this was not recorded within this setting and, therefore, were not extracted as part of the dataset in Phase Two. This inability to distinguish between inaccessible and unassessed data further complicates these findings. Evidence suggests that higher reports of *Cannot Assess* items within the IPOS are linked to declines in functionality [48]. Future research should explore these dynamics. Those involved in completing PROMs require ongoing support in understanding the roles these measures can play in identifying person-centred perspectives of need, informing both immediate care and changes over time for service impact. Providers should also consider extending support and training to professionals involved in psycho-social care. Broader approaches to addressing needs through PROM use in partnerships with other community-specific services and voluntary providers should also be explored [10].

All services supporting individuals over 65 should actively identify and recognise frailty within their population [49, 50]. A proactive approach can lead to timely, tailored decisions for the individual and contextualise the use of other clinical measures, alongside PROMs. The wide variety of tools for assessing frailty may make international comparisons challenging; however, globally, the CFS is increasingly adopted due to its simplicity and recommended for those with frailty in the UK [49, 51]. While preliminary evidence suggests that the CFS and AKPS can be translatable in palliative care settings [52], frailty-specific measures should be considered distinct, with appropriate training and support for their implementation.

## Strengths and limitations

Studies related to implementation are often context-specific. This study was conducted with a single specialist palliative care provider; thus, findings are not reproducible and may not be generalised. The significance of this work lies in how it has utilised the CFIR to support data integration to develop the programme theory, which provides insight for other settings considering implementing PROMs with those caring for older people with frailty and how PROMs might best capture person-centred needs and priorities. Older people with frailty are less likely to receive specialist palliative care [5]. Thus, action is required to address these challenges of using PROMs where older people with frailty are most likely to receive care and, indeed, where PROMs are currently less commonly used. Most of the PROMs in this study were staff-administered, deviating from how most other PROMs are used. A strength of this study was trying to understand PROM use in real-world settings outside of research. There are, however, some limitations to note. The service-users within the non-participatory observations were all White British. This did not reflect the ethnic diversity observed in the case-note review and may have impacted the use of the PROM. Methods such as non-participatory observations may have changed the behaviours of participants, whereby the completion of PROMs may have been altered. However, these were used within a mixed methods approach, and the researcher spent time building trust with staff through attending meetings and shadowing staff, which was considered to mitigate this potential bias. Although the data extraction period was a calendar year, future studies should include longer timeframes of data collection to avoid data censure due to the follow-up period. Furthermore, due to the level of missing data for the CFS, the severity of frailty was possibly underestimated in this study.

## Conclusion

This study demonstrates that PROMs can be effectively used with older adults living with frailty in the last phase of life, providing valuable insights into their implementation in community care settings where these individuals typically receive support. By utilising the CFIR and a logic model, the study highlights the complexities and variability involved in using PROMs with this population and the need for tailored context-appropriate measures. Existing PROMs and PREMs may need adaptation towards a capability approach better aligned with the priorities and experiences of older adults with frailty. Considering the importance placed upon data that PROMs and PREMs generate, greater emphasis should be placed on supporting those completing PROMs and ensuring that the priorities of older people with frailty are measured and actively used to inform and improve care decisions for this population.

## Supplementary Information

Below is the link to the electronic supplementary material.

Supplementary Material 1

## Data Availability

Access to supporting data should be made through the corresponding author. Due to the inclusion of clinical data, access is not openly available.

## Abbreviations

OPWSF: Older People with Severe Frailty
HCP: Healthcare Professional
PROM: Patient Reported Outcome Measure
PREM: Patient Reported Experience Measure
CFIR: Consolidated Framework for Implementation Research
MRC: Medical Research Council
IPOS: Integrated Palliative Care Scale
AKPS: Australia-modified Karnofsky Performance Status
CFS: Clinical Frailty Scale
