# Supplementary material for: Developing a programme theory of implementing patient-reported outcome measures for older people living with severe frailty: a mixed methods study using the consolidated framework for implementation research
Source: J Patient Rep Outcomes. 2025 Oct 24;9:126. doi: 10.1186/s41687-025-00951-9 (PMC12552205; doi:10.1186/s41687-025-00951-9)
Supplement: Supplementary file 1 — Supplementary Material 1 [file 41687_2025_951_MOESM1_ESM.docx]

### Supplementary data file 1: Observational Proforma

PROFrail: Observational study

| Date and Time: |  | Observation ID: |  |
| --- | --- | --- | --- |
| Site ID: |  | Pr ID: |  |
| Phase of illness: AKPS: |  | Clinical frailty Scale (CFS) score |  |
| Reason for episode of care |  | | |

## Before visit:

### Professional participant characteristics

| Professional Title/role |  |
| --- | --- |
| Experience |  |

| Site ID |  |
| --- | --- |
| Service ID |  |
| Place of episode of care |  |

### Older adult characteristics:

| First assessment | Yes No |
| --- | --- |
| Accommodation type |  |
| Ethnicity Code |  |
| Gender of patient |  |

### Caregiver(s) participant characteristic:

| How many caregivers present? |  | | |
| --- | --- | --- | --- |
| Wife  Husband  Daughter |  | Son  Sibling  Friend  Other (detail) |  |

## During Observation: Time at start of visit: Time at end of visit:

| Domain  Initiated by: (Pr, OA, C ); Time spent (e.g., 12:00 to 12:10) |
| --- |
| *Physical*  ADL  Functioning  Physical symptoms  Medication |
| *Psychological*  Anxiety  Depression  Self-worth  Adjustment |
| *Social*  Social network  Care Network |
| *Spiritual*  Loss of hope  Connectedness to  existence |
| *Practical*  Environmental  Information/ financial  Care planning  Individualised care |
| *Other observations:* |

## After visit summary:

| PROM Name: |  |
| --- | --- |
| PROM completed: | Before During After |
| PROM tool or discussed during visit: | YES/NO |
| Completed By: | Pr OA C |
| Completed items: | 1—2—3—4—6—7—8—9--10--Free text |

### Researcher’s thoughts of visit:

| Overall summary |  |
| --- | --- |
| What role do PROMs and/or PREMs have within the consultation? |  |
| Were there any challenges of PROM completion? |  |

### Was what mattered most to older adult with frailty captured within measure?

|  |
| --- |

### Was what mattered most to caregiver captured within measure?

|  |
| --- |

### Supplementary data file 2: Topic guide

## Introduction

*“The focus of today’s interview is around your thoughts and experiences on using Clinical tools such as IPOS used within your clinical practice and how these particularly relate to assessment and care provided to those with advancing frailty.”*

1. *Before I carry on are you happy with me using the term* ***IPOS***
2. *What does the term* ***frailty*** *mean to you when thinking about different patient groups?*
3. Could you talk through how and when you use the *IPOS/How are you questionnaire^[[1]](#footnote-1)^* and similar tools within your current practice right now?
   - How else are they used within the wide practice at St Christopher’s
   - How are the used along side other measures

*(Aim: Gain a broad understanding of how PROMs are used within setting and practice)*

Prompts:

WHAT - Which measure is used

WHO - completes the measure

WHEN - are the measures completed - before, during or after the consultation? Frequency

WHY – are there particular times when this is done more often?

1. Could you talk about benefit and challenges you find when a *IPOS/How are you questionnaire* with/for the patient

(Aim: *Gain a broad understanding of how professional views the role of PROMs within their practice and provision of care*)

Probes:

Identification of need

Monitor changes

Does the use of IPOS assist discussions with patient?

Does the IPOS act as an Aid memoir?

Communication with colleagues

Evidencing care/documentation of care

Supporting the services to evidence quality of care

Are the challenges using the tool/scales within the tool?

Are the challenges with integration with other assessments/IT

Impact on next episode of care?

*The next set of questions relate to your views and experience of using IPOS with older people with frailty within your caseload.*

1. In you experience, can you describe how completing a *IPOS/How are you questionnaire* with an older adult with frailty might compares to another patient groups?

*(Aim: Are they any challenges or issues that are unique to this group)*

Prompts:

Practical challenges.

Engagement challenges

Capturing need

Role of the Caregiver in completing the measure

*So I’d like you to think about one of your patients*

1. can you describe how their care needs were captured with the *IPOS/How are you questionnaire* that are currently used?

*(Aim: Identify missing areas of need that are not captured within tool AND identify redundant items)*

Prompts:

Is there anything missing?

Is there anything that doesn’t relate to this patient group

1. Are there questions within the *IPOS/How are you questionnaire* that are find difficult to ask or address with older people with frailty

Why?

*(Aim: Identify barriers to PROM completion)*

Prompts:

These might be Professional difficulties – Not wanting to ask/skills/training/experience/relevancy

Engagement difficulties from patient – Not wanting to answer

Does this differ/is the same or same with other groups

1. When a caregiver is present, how well do you feel that they are involved in capturing the needs within these tools?

*(Aim: Identify missing areas of need that are not captured within tool)*

Prompts:

Is there anything missing?

1. What part of the tools do you find most useful with this group */How are you questionnaire* with this group?
   - Do you ever use the Views on care questions
2. What would improve the use of *IPOS/How are you questionnaire* with this group?

*(Aim: Identify enablers and also missing areas of need that are not captured within tool)*

Prompts:

Is there anything missing/that doesn’t relate

Are there ways to address barriers

1. Ending the interview

That is all the questions that I wanted to ask, but:

- anything you would like to add
- is there anything else you thought would have been asked in interview or would like to ask me

## Supplementary data file 3

### Case-Note review - Extracted data points

| **Demographic details:**   - Age - Gender - Ethnicity - Diagnosis - Postcode (used as a proxy for index of deprivation) - Referral source - Lives alone (Y/N) - Date of referral - Date for discharge |
| --- |
| **Outcome measurements extracted:**   - Clinical Frailty Scale (CFS) (Rockwood, 2005) - Phase of illness (Masso et al., 2015) - Australian Karnofsky Performance scale (AKPS) (Abernethy et al., 2005a) - Integrated Palliative outcome scale (IPOS) (Murtagh et al., 2019). |
| **Additional items extracted in Sub-sample:**   - Textual notes associated with three additional qualitative data points routinely asked at the beginning of the assessment:   - *“What is important to you right now”*   - “*What would you like to achieve in the next short while*”   - “What are your main concerns” - A PREM: *Views-on-care* (Addington-Hall et al., 2014).  These questions and items were included, alongside the IPOS, in the How are you form given to patients in the study setting. |

| Supplementary data file 4: The CFIR (Damschroder et al., 2022) **Framework Guidance:** The CFIR is intended to be used to collect data from individuals who have power and/or influence over implementation outcomes. See the CFIR Outcomes Addendum for guidance on identifying these individuals and selecting outcomes.  The CFIR must be fully operationalized prior to use in a project:  1) Define the subject of each domain for the project (see guidance for each domain below). 2) Replace broad construct language with project-specific language if needed. 3) Add constructs to capture salient themes not included in the updated CFIR. | |
| --- | --- |
| **I. INNOVATION DOMAIN Innovation: The “thing” being implemented, e.g., a new clinical treatment, educational program, or city service.  [Document the innovation being implemented, e.g., innovation type, innovation core vs. adaptable components, using a published reporting guideline. Distinguish the innovation (the “thing” that continues when implementation is complete) from the implementation process and strategies used to implement the innovation (activities that end after implementation is complete).]** | |
| **Construct Name** | **Construct Definition** *The degree to which:* |
| A. Innovation Source | The group that developed and/or visibly sponsored use of the innovation is reputable, credible, and/or trustable. |
| B. Innovation Evidence-Base | The innovation has robust evidence supporting its effectiveness. |
| C. Innovation Relative Advantage | The innovation is better than other available innovations or current practice. |
| D. Innovation Adaptability | The innovation can be modified, tailored, or refined to fit local context or needs. |
| E. Innovation Trialability | The innovation can be tested or piloted on a small scale and undone. |
| F. Innovation Complexity | The innovation is complicated, which may be reflected by its scope and/or the nature and number of connections and steps. |
| G. Innovation Design | The innovation is well designed and packaged, including how it is assembled, bundled, and presented. |
| H. Innovation Cost | The innovation purchase and operating costs are affordable. |
| **II. OUTER SETTING DOMAIN Outer Setting: The setting in which the Inner Setting exists, e.g., hospital system, school district, state. There may be multiple Outer Settings and/or multiple levels within the Outer Setting (e.g., community, system, state). Project Outer Setting(s): [Document the actual Outer Setting in the project, e.g., type, location, and the boundary between the Outer Setting and the Inner Setting.]** | |
| **Construct Name** | **Construct Definition** *The degree to which:* |
| A. Critical Incidents | Large-scale and/or unanticipated events disrupt implementation and/or delivery of the innovation. |
| B. Local Attitudes | Sociocultural values (e.g., shared responsibility in helping recipients) and beliefs (e.g., convictions about the worthiness of recipients) encourage the Outer Setting to support implementation and/or delivery of the innovation. |
| C. Local Conditions | Economic, environmental, political, and/or technological conditions enable the Outer Setting to support implementation and/or delivery of the innovation. |
| D. Partnerships & Connections | The Inner Setting is networked with external entities, including referral networks, academic affiliations, and professional organization networks. |
| E. Policies & Laws | Legislation, regulations, professional group guidelines and recommendations, or accreditation standards support implementation and/or delivery of the innovation. |
| F. Financing | Funding from external entities (e.g., grants, reimbursement) is available to implement and/or deliver the innovation. |
| G. External Pressure | External pressures drive implementation and/or delivery of the innovation. Note: Use this construct to capture themes related to External Pressures that are not included in the subconstructs below. |
| 1. Societal Pressure | Mass media campaigns, advocacy groups, or social movements or protests drive implementation and/or delivery of the innovation. |
| 2. Market Pressure | Competing with and/or imitating peer entities drives implementation and/or delivery of the innovation. |
| 3. Performance-Measurement Pressure | Quality or benchmarking metrics or established service goals drive implementation and/or delivery of the innovation. |
| **III. INNER SETTING DOMAIN Inner Setting: The setting in which the innovation is implemented, e.g., hospital, school, city. There may be multiple Inner Settings and/or multiple levels within the Inner Setting, e.g., unit, classroom, team. Project Inner Setting(s): [Document the actual Inner Setting in the project, e.g., type, location, and the boundary between the Outer Setting and the Inner Setting.]** | |
| **Construct Name** | **Construct Definition** *The degree to which:* |
| *Note:* | *Constructs A – D exist in the Inner Setting regardless of implementation and/or delivery of the innovation, i.e., they are persistent general characteristics of the Inner Setting.* |
| A. Structural Characteristics | Infrastructure components support functional performance of the Inner Setting. Note: Use this construct to capture themes related to Structural Characteristics that are not included in the subconstructs below. |
| 1. Physical Infrastructure | Layout and configuration of space and other tangible material features support functional performance of the Inner Setting. |
| 2. Information Technology Infrastructure | Technological systems for tele-communication, electronic documentation, and data storage, management, reporting, and analysis support functional performance of the Inner Setting. |
| 3. Work Infrastructure | Organization of tasks and responsibilities within and between individuals and teams, and general staffing levels, support functional performance of the Inner Setting. |
| B. Relational Connections | There are high quality formal and informal relationships, networks, and teams within and across Inner Setting boundaries (e.g., structural, professional). |
| C. Communications | There are high quality formal and informal information sharing practices within and across Inner Setting boundaries (e.g., structural, professional). |
| D. Culture | There are shared values, beliefs, and norms across the Inner Setting. Note: Use this construct to capture themes related to Culture that are not included in the subconstructs below. |
| 1. Human Equality-Centeredness | There are shared values, beliefs, and norms about the inherent equal worth and value of all human beings. |
| 2. Recipient-Centeredness | There are shared values, beliefs, and norms around caring, supporting, and addressing the needs and welfare of recipients. |
| 3. Deliverer-Centeredness | There are shared values, beliefs, and norms around caring, supporting, and addressing the needs and welfare of deliverers. |
| 4. Learning-Centeredness | There are shared values, beliefs, and norms around psychological safety, continual improvement, and using data to inform practice. |
| *Note:* | *Constructs E – K are specific to the implementation and/or delivery of the innovation****.*** |
| E. Tension for Change | The current situation is intolerable and needs to change. |
| F. Compatibility | The innovation fits with workflows, systems, and processes. |
| G. Relative Priority | Implementing and delivering the innovation is important compared to other initiatives. |
| H. Incentive Systems | Tangible and/or intangible incentives and rewards and/or disincentives and punishments support implementation and delivery of the innovation. |
| I. Mission Alignment | Implementing and delivering the innovation is in line with the overarching commitment, purpose, or goals in the Inner Setting. |
| J. Available Resources | Resources are available to implement and deliver the innovation. Note: Use this construct to capture themes related to Available Resources that are not included in the subconstructs below. |
| 1. Funding | Funding is available to implement and deliver the innovation. |
| 2. Space | Physical space is available to implement and deliver the innovation. |
| 3. Materials & Equipment | Supplies are available to implement and deliver the innovation. |
| K. Access to Knowledge & Information | Guidance and/or training is accessible to implement and deliver the innovation. |
| **IV. INDIVIDUALS DOMAIN Individuals: The roles and characteristics of individuals.** | |
| **ROLES SUBDOMAIN Project Roles: [Document the roles applicable to the project and their location in the Inner or Outer Setting.]** | |
| **Construct Name** | **Construct Definition** |
| A. High-level Leaders | Individuals with a high level of authority, including key decision-makers, executive leaders, or directors. |
| B. Mid-level Leaders | Individuals with a moderate level of authority, including leaders supervised by a high-level leader and who supervise others. |
| C. Opinion Leaders | Individuals with informal influence on the attitudes and behaviors of others. |
| D. Implementation Facilitators | Individuals with subject matter expertise who assist, coach, or support implementation. |
| E. Implementation Leads | Individuals who lead efforts to implement the innovation. |
| F. Implementation Team Members | Individuals who collaborate with and support the Implementation Leads to implement the innovation, ideally including Innovation Deliverers and Recipients. |
| G. Other Implementation Support | Individuals who support the Implementation Leads and/or Implementation Team Members to implement the innovation. |
| H. Innovation Deliverers | Individuals who are directly or indirectly delivering the innovation. |
| I. Innovation Recipients | Individuals who are directly or indirectly receiving the innovation. |
| **CHARACTERISTICS SUBDOMAIN Project Characteristics: [Document the characteristics applicable to the roles in the project based on the COM-B system or role-specific theories.]** | |
| **Construct Name** | **Construct Definition:** *The degree to which:* |
| A. Need | The individual(s) has deficits related to survival, well-being, or personal fulfillment, which will be addressed by implementation and/or delivery of the innovation. |
| B. Capability | The individual(s) has interpersonal competence, knowledge, and skills to fulfill Role. |
| C. Opportunity | The individual(s) has availability, scope, and power to fulfill Role. |
| D. Motivation | The individual(s) is committed to fulfilling Role. |
| **V. IMPLEMENTATION PROCESS DOMAIN  Implementation Process: The activities and strategies used to implement the innovation. Project Implementation Process: [Document the implementation process framework and/or activities and strategies being used to implement the innovation. Distinguish the implementation process used to implement the innovation (activities that end after implementation is complete) from the innovation (the “thing” that continues when implementation is complete).]** | |
| **Construct Name** | **Construct Definition** *The degree to which individuals:* |
| A. Teaming | Join together, intentionally coordinating and collaborating on interdependent tasks, to implement the innovation. |
| B. Assessing Needs | Collect information about priorities, preferences, and needs of people. Note: Use this construct to capture themes related to Assessing Needs that are not included in the subconstructs below. |
| 1. Innovation Deliverers | Collect information about the priorities, preferences, and needs of deliverers to guide implementation and delivery of the innovation. |
| 2. Innovation Recipients | Collect information about the priorities, preferences, and needs of recipients to guide implementation and delivery of the innovation. |
| C. Assessing Context | Collect information to identify and appraise barriers and facilitators to implementation and delivery of the innovation. |
| D. Planning | Identify roles and responsibilities, outline specific steps and milestones, and define goals and measures for implementation success in advance. |
| E. Tailoring Strategies | Choose and operationalize implementation strategies to address barriers, leverage facilitators, and fit context. |
| F. Engaging | Attract and encourage participation in implementation and/or the innovation. Note: Use this construct to capture themes related to Engaging that are not included in the subconstructs below. |
| 1. Innovation Deliverers | Attract and encourage deliverers to serve on the implementation team and/or to deliver the innovation. |
| 2. Innovation Recipients | Attract and encourage recipients to serve on the implementation team and/or participate in the innovation. |
| G. Doing | Implement in small steps, tests, or cycles of change to trial and cumulatively optimize delivery of the innovation. |
| H. Reflecting & Evaluating | Collect and discuss quantitative and qualitative information about the success of implementation. Note: Use this construct to capture themes related to Reflecting & Evaluating that are not included in the subconstructs below. |
| 1. Implementation | Collect and discuss quantitative and qualitive information about the success of implementation. |
| 2. Innovation | Collect and discuss quantitative and qualitative information about the success of the innovation. |
| I. Adapting | Modify the innovation and/or the Inner Setting for optimal fit and integration into work processes. |

### Supplementary data file 5:

### Good reporting of a mixed-method study (GRAMMs) Checklist

| Guideline | Page Information |
| --- | --- |
| 1. Describe the justification for using a mixed methods approach to the research question | Pg 5 line 101 |
| 2. Describe the design in terms of the purpose, priority and sequence of methods | Pg 4 lines 94-99 |
| 3. Describe each method in terms of sampling, data collection and analysis | Pg 5 lines 124-Pg6 lines 165 |
| 4. Describe where integration has occurred, how it has occurred and who has participated in it | Pg 6 line 178 to page 7 line 192 |
| 5. Describe any limitation of one method associated with the present of the other method | Pg 23 lines 461-465 |
| 6. Describe any insights gained from mixing or integrating methods | Pg 23 lines 452-253 |

Reference:

O'Cathain A, Murphy E, Nicholl J. The quality of mixed methods studies in health services research. J Health Serv Res Policy. 2008;13: 92-98

### Supplementary data file 6: Mapping data to components of CFIR

Data source:
1 – Interview

2 - Observation

3 - Casenote review

| CFIR Domain | Summary of findings | Data |
| --- | --- | --- |
| Innovation:  The “thing” being implemented | Use of PROM that does not address all the needs of Older people with frailty (-, 1a,1b ) | - *"The difficulty is that there's a bit more to people with frailty than it doesn't do and it's where do you put that information in you know "In2p2* |
|  | Variability in use of available PROMs (-/+, 1a, 1b, 2) | - *"I'm probably guilty of doing more staff-led one, perhaps. Often due to the lack of preparation on my part" IN9P9* - Findings of the Case note review identified that there was a greater number of missing items in the psycho-social domain compared to items in the physical domain |
| Process:  How innovation is implemented | When patient completed, involves others (+/-, 1a, 1b) | - *"Typically with our older people with frailty, there's more deferment should we say to family members... I don't know if they struggle more, but knowing quite how to elaborate on it when they ask you without deviating from what the question is saying and what is the right language to use to support people to answer it themselves and appropriately without steering. I think that can be a challenge."In1P1* - *"But perhaps our elderly frail patients, its often someone else filling it out for them or we’re asking the questions to them."In4P4* - Findings from the observations found formal and informal caregiver involved in assessments |
|  | Lack of shared understanding of the role of PROM in assessment (-, 1a, 1b) | - *"The amount of times people don't understand know why I'm doing this thing. "In1P1* - *"We could use the tool, the tool could be used better if the bit that came before it was a bit better, improved"in2p2* - *"9 times out of 10 its not done. “ they say, I’ve got a bunch of paper work, I haven’t looked at it. Who are you. ”In11P11* - *"Often people have said like, "why? Why all the questions?"... which obviously for us is like trying to get information”In3P3* |
|  | Experience items are rarely used (- , 1a, 1b) | - *"I don't think it [View on Care] give's me any additional things when I'm doing the assessment and is burdensome to people so I don’t do it."P1In1* - *"I’ve never do it [View on Care], because people will just talk about the external care that they are getting from the carers and the care."In5In5* - *"So if I am doing the IPOS with somebody then I'm not doing it at all. I'm not asking them their views on care... then I'm crossing that bit off and saying "just fill it up to there for now.""In10P10* - View on care items were not found to be used within any observations |
|  | Majority proxy completed (+/-, 1a, 1b,2,)  Inconsistent use of CFS alongside PROMs (-, 1b,2) | 7 out of 10 in observation were proxy completed and 1^st^ IPOS completed by a professional in 237 out of 441 cases  Observations and case note review found CFS was not used consistently across caseload compared to AKPS |
| Needs of the Older person with frailty | Heterogeneity in needs (-/+, 1a,1b) | - *"a lot of the ipos that may not actually be relevant because, because a lot of it's like pain, nausea, vomiting bowels. And all those kind of things, which obviously someone could still present that way, but without a kind of without a diagnosis of cancer" In3p3* - *"I think there’s possible missing um, more of the social aspect, that’s quite a large part of our assessment and a lot of more frail people have quite demanding kind of social needs."In6P6* - Observation identified that concerns related to practical care a key focus and concern |
|  | Patient burden - Cognitive and physical capabilities (-, 1a,1b) | - *And the elderly frail do not want to, they are feeling awful... You have to concentrate on which part of the IPOS you think would be relevant and important to find out. " In5P* - *"Maybe they can’t hear or see as well, they are more willing for somebody else to do it on their behalf instead of them actually doing it." In8p8* |
|  | Accessing care in crisis (-1b,2) | - Case note review indicating older people with frailty were typically referred to service in deteriorating condition and poor functionality |
|  | Caregivers come with own set of needs (-/+,1b,2) | Observation and in-depth case note sub-sample identified that caregiver support was a key focus in assessment and care. |
| Individuals: The roles and characteristics of individuals | What matters conversations most valued by staff (+, 1a,1b) | - *"The most important question? what matters to you the most? And that gives you really, really surprised answer. And I think when we ask about what is the most important for you and we try to do something to match that needs is when you are helping them to be in peace with themselves as well." In7P7* |
|  | Value of soft assessment skills (+/-, 1a,1b) | - *You going to get an idea of what is going on, but with an elders frail, the only way you’re actually going to find out what going on is by talking to them and trying to find out, what has changed, yeah, what’s important to you, what do you want to achieve" In5p5* - *"I respect a lot of the IPOS... but I'm saying it's a guide and I think we take the risk to be very attached to the guide unless open to catch What is important for that patient and what are the needs and what are the worries and how we can help them rather than to be super attached to the to the paper."In7p7* |
|  | Challenges in assessing psychosocial/spiritual needs with PROM (-, 1a,) | - *"I think the psychological [items] are harder to score. You don't want to say someone isn't having any, you know anxiety or things if they are, but it's not particularly obvious in their behaviour and they haven’t mentioned it, I think people less, can be less, open to saying how they feel so psychologically. And I've had a, you know, quite a few older gentleman, particularly really struggle with the, the, second half of it, the psychological bit, not knowing what to put."In1P1* - "I think the psychological one is I always find challenging… it's probably something I'm, you know, less familiar with, you know less so it's not that that I can't do it. I think it's that. It's always so if you're asking an older, older person about some of the you know, they're not often very resistant about you're poking about in their psyche."In2P2 ” |
|  | Concern of a dependence on PROMs as tickbox exercise can miss WMM (-, 1a) | - *"I guess on of the kind of downfalls it can feel a bit like a check list. “tell me your pain, tell me if you’re breathless” That kinda, I perhaps I find that kind of a bit, some people are like, they don’t just want to be asked they’d rather be having a fluid conversation" In4P4* - *"So, I think if you’re sitting down with a piece of paper, you’re only focused on that piece of paper you not doing a rapport with patients."In5P5* - *"But sometimes its you know “I need help with talking to my daughter about ceilings of treatment.” That’s something that you can’t capture in a tick box. "In11P11* |
| Inner Setting: The setting in which the innovation is implemented | Goal based values (+, 1a,1b) | - *"...we're are very goal-based here aren’t we. I think its good." In3P3* |
|  | PROMs used across organisation (+, 1a,1b) | - *"it's a universal tool that we're all using doctors, nurses, everyone in the kind of organisation"In3P3* - *"And also if you are learning to be a nurse in palliative care, that will be your your Bible." In7P7,* - *"I think we teach junior members of staff to frame their assessments around the How are you form because its quite comprehensive"In11P11* - *The PROM was used as screening tool, communicate between colleagues and structure documentation within the observation* |
|  | PROM used to communicate within teams (+ 1a,1b, 2) | - *"The IPOS has the same approach and that’s really important when their going to speak to me on the phone and then their gonna not speak to me again because they only speak to me once. And then someone else is gonna, so actually, all, if we are all, all of us using the same approach, you know the fact that he'd already filled out the IPOS form, that kinda of, I think it just, kind of helped."IN4P4* - *The PROM was used to discuss individuals with colleagues within the observation* |
|  | Workforce Pressures (-, 1a) | - *"There is a high turnover of staff... I don’t think its routinely retaught. So people are using it, but they are using it how they think it should be used, rather than how it definitely should be used... Its being used well, but maybe not perfectly." In11P11* - *"I think we all need encouragement in that, we need to use it as it's considered to be our bog standard baselines sort of tool" In2P2* |

1. IPOS and How are you questionnaire are the names of patient reported outcome measures which are used within the clinical setting the interviews will be taking place and the name which will be most familiar to the participants [↑](#footnote-ref-1)
